# Early functional results after Hemiarthroplasty for femoral neck fracture: a randomized comparison between a minimal invasive and a conventional approach

**DOI:** 10.1186/1471-2474-13-141

**Published:** 2012-08-08

**Authors:** Felix Renken, Svenja Renken, Andreas Paech, Michael Wenzl, Andreas Unger, Arndt P Schulz

**Affiliations:** 1 University Hospital Schleswig Holstein, Campus Lübeck, Ratzeburger Allee 160, D 23538, Lübeck, Germany; 2 University Lübeck, Medical Faculty, Lübeck, Germany; 3 Klinikum Ingolstadt, Ingolstadt, Germany; 4 BG Trauma Hospital Hamburg, Bergedorfer Strasse, Hamburg, Germany

## Abstract

**Background:**

A minimal invasive approach for elective hip surgery has been implemented in our institution in the past. It is widely hypothesized that implanting artificial hips in a minimal invasive fashion decreases surgical trauma and is helpful in the rehabilitation process in elective hip surgery. Thereby geriatric patients requiring emergency hip surgery also could theoretically benefit from a procedure that involves less tissue trauma.

**Methods:**

Sixty patients who sustained a fractured neck of femur were randomly assigned into two groups. In the minimal invasive arm, the so called “direct anterior approach” (DAA) was chosen, in the conventional arm the Watson-Jones-Approach was used for implantation of a bipolar hemi-arthroplasty.

Primary outcome parameter was the mobility as measured by the four-item-Barthel index. Secondary outcome parameters included pain, haemoglobin-levels, complications, duration of surgery, administration of blood transfusion and external length of incision. Radiographs were evaluated.

**Results:**

A statistically significant difference (p = 0,009) regarding the mobility as measured with the four-item Barthel index was found at the 5th postoperative day, favouring the DAA. Evaluation of the intensity of pain with a visual analogue scale (VAS) showed a statistically significant difference (p = 0,035) at day 16. No difference was evident in the comparison of radiographic results.

**Conclusions:**

Comparing two different approaches to the hip joint for the implantation of a bipolar hemi-arthroplasty after fractured neck of femur, it can be stated that mobilization status is improved for the DAA compared to the WJA when measured by the four-item Barthel index, there is less pain as measured using the VAS. There is no radiographic evidence that a minimal invasive technique leads to inferior implant position.

Level of Evidence: Level II therapeutic study.

## Background

The incidence of hip fractures related to osteoporosis is steadily increasing [1,2]. In Germany, a rise of 74% in the incidence of proximal femoral fractures until the year 2020 is forecasted [2]. In the same study, a current in-hospital mortality of 8.6% was described in over 85 year old patients. The morbidity and mortality after this kind of fracture is thereby high [2,3]. The risk factors for mortality include the development of one or more postoperative complications [4]. These include the development of chest infections, deep vein thrombosis, muscle wasting and pressure sores [5-7]. An early and consequent postoperative mobilization should therefore decrease morbidity and mortality. Measuring the influence of an intervention regarding mortality and morbidity is extremely complex in hip arthroplasty as it requires enormous case numbers [8], these numbers can often only be gained in large multicentre studies which are very complex, expensive and difficult to set up. As it has been described in the past that morbidity and mortality in elderly patients with a fractured neck of femur can be positively influenced by early mobilization and a high quality of care [9-13], we designed a study that solely measures the mobilization process.

The effects of elective minimal invasive (MIS) hip arthroplasty have been widely researched. So far, the only certain evidence is that there is a positive cosmetic effect [14]. A meta-analysis could determine a reduced blood loss [15]. In the same study, a significant difference in clinical outcome parameter (in this study the Harris Hip Score, HHS) could not be found. Another meta-analysis also failed to detect differences regarding the HHS [16], although there was a detectable trend towards better HHS score results for MIS procedures. Another study has described a better pain control and earlier hospital discharge; more MIS patients were using just a single assistive device at the time of discharge [17].

It remains unclear if the test methods that are used to detect the differences between minimal invasive and conventional elective hip arthroplasty (often HHS or WOMAC) might lack the discriminatory power when it comes to emergency procedures in a geriatric patient population. It is certain that these test methods are of limited use in a geriatric population. In a current systemic literature review, 14 commonly used outcome scales were found used for patients with proximal femoral fractures, furthermore 43 additional scales not in common usage [18,19]. None of these were validated for use in this patient group. The author concluded that mobility and disease specific scales should be considered appropriate until validation and consensus recommendations are available.

The Barthel index [20], one of the above mentioned scales, is a widely used scale that measures activities of daily ling (ADL) in a geriatric population. It is not disease specific but has also been used for patients after proximal femoral fractures [21-26].

For the purpose of disease specific use in clinical trials, epidemiological studies, and audit, a short form Barthel index containing 3,4 or all of the items transfers, bathing, toilet use, stairs, and mobility has been examined regarding acceptability, reliability, validity, and responsiveness in the past [27].

The aim of this study was to compare the rehabilitation process regarding mobilization for patients that had a femoral neck fracture treated by bipolar hemi-arthroplasty either via a MIS procedure or a conventional surgical access. To measure this, we used a 4-item abbreviation of the Barthel index, focusing on lower extremity motoric function.

### Primary outcome criterium

Mobilization status as assessed by the 4-item abbreviation of the Barthel index, focusing on lower extremity motoric function.

### Secondary outcome criteria

Comparison of blood loss by measurement of required units of packed red cells.

Comparison of postoperative pain as determined by the visual analogue scale (VAS) [28].

Comparison of medical and surgical complications in the first 40 days from surgery.

Comparison of radiographic results regarding implant position, femoral offset and leg length.

### Hypothesis

Patients suffering of an osteoporosis related femoral neck fracture can be faster mobilized and regain their motoric status measured with a four-item-Barthel index if the injury is treated via a minimal invasive direct anterior approach when compared to a conventional approach.

## Methods

### Study design

A consecutive series of patients with a fractured neck of femur were prospectively randomized into two groups on the day of admission. In group I (study group), the intervention consisted of a direct anterior approach to the hip [14,29,30] with implantation of a bipolar hemi-endoprosthesis using specific surgical tools. In group II (control group), a Watson-Jones approach [31] was chosen for the implantation of the same type of endoprosthesis with conventional tools.

Inclusion criteria:

These included all patients with an indication for bipolar hemi-endoprosthesis of the hip for proximal femoral fractures (Garden 3 and 4 fractures [32]), an age above 60, the ability to give informed consent or the availability of a court ordered legal guardian. After ethical committee discussion and approval, patients that were not able to perform informed consent were included, if there was a court ordered carer in the area of medical care and consenting, and this person consented to the trial.

Exclusion criteria were active infection (systemic or local), history of infection in the injured hip joint, immobility, current treatment for malignant disease, suspicion of so far undiagnosed malignant disease, rheumatoid arthritis, neurological deficits of the lower extremity including Parkinsons disease and skin diseases in the area of proposed incisions.

**Figure 1:**
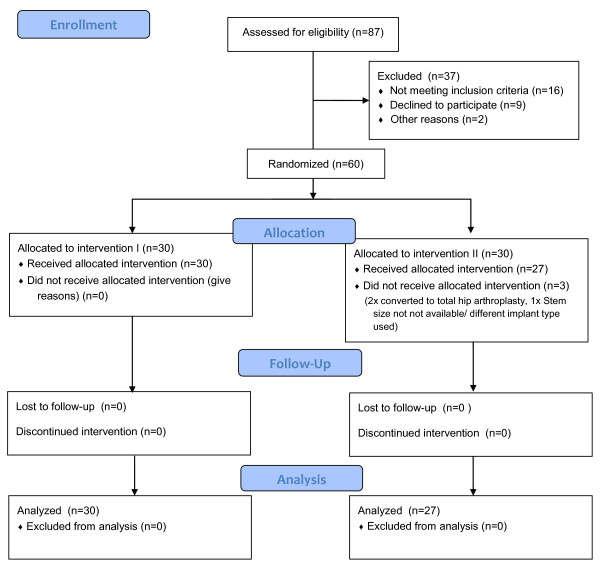
**Study diagram.** CONSORT Flowchart of enrolment and allocation to groups 1 and 2.

In group I, 30 patients completed follow up and were analysed. In group II it was twice necessary to convert to total hip arthroplasty (THA) due to immediate dislocation. In one case an implant of a different manufacturer was used because of a missing size. In this group, 27 patients were included in the data analysis (see Flowchart in Figure 1).

### Study protocol

The study protocol and consent form met the approval of the local ethical committee. The study is registered in the German clinical trials register (DRKS Number: DRKS00003332). Study centre was a large University Hospital treating approximately 250 proximal femoral fractures per annum. Three senior surgeons that were well skilled to perform a Watson-Jones approach (WJA) underwent thorough cadaver training in the DAA technique. After that, each of them performed a minimum of 20 procedures before the trial.

### Patients

In group I (DAA group), 30 patients were analysed, in group II it were 27 (for details of recruitment see diagram 1). Statistical evaluation found no significant difference between the cohorts regarding sex, age, ASA grade, body-mass index (BMI) and other demographic data (see Table 1).

**Table 1:** Demographic data

**Age**	**Minimum**	**Median**	**Maximum**	**Std.Dev.**	**Significance-level**
***DAA***	70	84	94	5,8	
**WJA**	71	87,5	96	7,0	n.s.
***BMI***					
**DAA**	23	26	27	1,4	
**WJA**	17,3	21,5	27	2,6	n.s.
***Sex***	Female	Male			Significance-niveau
**DAA**	26	4			
**WJA**	24	3			n.s.
**Hemoglobin preop. g/l**	Minimum	Median	Maximum	Std.Dev.	Significance-niveau
DAA	98,0	127,5	150,0	13,4	
WJA	89,0	126,0	159,0	16,8	n.s.

Demographic data with statistical comparison of the two cohorts “direct anterior approach, DAA” and ” Watson Jones approach, WJA”. n.s. = not significant.

### Methods

Surgical technique DAA: The technique has been described in detail elsewhere [14,29,33-36]. Similar to the WJA, the DAA procedure is performed in a supine position. The skin incision starts 2–3 cm distal and lateral to the anterior superior iliac spine along the medial border of the M. tensor fascia lata. This approach uses an inter-muscular and inter-nerval plane between the sartorius, rectus femoris, and tensor fasciae latae [34]. For the femoral shaft preparation specific retractors and broach handles [37] where used (Stryker MIS Set, Duisburg, Germany).

**Figure 2:**
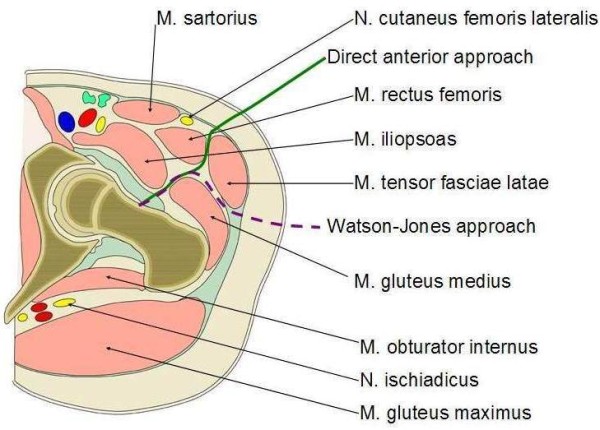
**Surgical access anatomy.** Comparison of access path with DAA (−−−) versus WJA (− − −).

The antero-lateral approach initially described by Watson-Jones [31] is well published. The main differences regarding the surgical anatomy are illustrated in Figure 2. In both groups, parenteral single shot antibiosis was administered and a deep wound drain was used. Postoperatively, 2 units of packed red cells were administered when a haemoglobin level of <80 g/l was detected.

### Implants

In all cases, a cemented anatomical ABG® II stem was used (Stryker, Duisburg, Germany) with 40 g of PMMA-bone cement with Gentamicin (Palacos-RG, Heraeus Medical GmbH, Wehrheim, Germany). The bipolar head used was a UHR® (Universal Head Bipolar System, Stryker, Duisburg, Germany) in all cases.

### Radiographs

Standardised views of the pelvis a.p. and an axial view were taken at the second postoperative day. The femoral offset was measured as the perpendicular distance of the femoral shaft axis and the rotational center of the hip joint. Indirect leg lenght measure was defined as the distance of the tip of the trochanter minor to a diagonal line at the ischiac tubercle [38]. The inured site was compared to the contralateral site.

The alignment was determined in comparison of the axis of the femoral shaft in comparison to the longitudinal axis of the prosthesis [39]. Results were notes as “normal”, “varus” or “valgus” alignment. Radiographs were evaluated by an independent radiologist who was blinded to the surgical method used.

### Data management and statistical analysis

Data acquisition was by two physicians unrelated to the surgical team. The data were transferred to an electronic database (IBM SPSS, Version 16, Chicago, USA) twice by two independent operators. A randomization list was created with an external generator (Random.org, Dublin, Ireland) using atmospheric noise. The randomization was via dark blue envelopes opened shortly before surgery.

The preoperative data included: age, sex, height, weight, body mass index (BMI), four item Barthel index with the scoring system as described by Granger [40] (Table 2), ASA grade as defined by anesthesiologist, VAS score [28] and laboratory parameter.

**Table 2:** Modified Barthel score

**Item**	**Score**
DRESSING	0 = dependent
	5 = needs help but can do about half unaided
	10 = independent (including buttons, zips, laces, etc.)
TOILET USE	0 = dependent
	5 = needs some help, but can do something alone
	10 = independent (on and off, dressing, wiping)
TRANSFERS (BED TO CHAIR AND BACK)	0 = unable, no sitting balance
	5 = major help (one or two people, physical), can sit
	10 = minor help (verbal or physical)
	15 = independent
MOBILITY (ON LEVEL SURFACES)	0 = immobile or < 50 yards
	5 = wheelchair independent, including corners, > 50 yards
	10 = walks with help of one person (verbal or physical) > 50 yards
	15 = independent (but may use any aid; for example, stick) > 50 yards
Max. Score	50 Points

The modified 4-item Barthel Index (Mahoney FI, 1965).

The intra-operative dataset included the procedure time from skin incision to skin suture, length of dermal incision, complications and used implants.

The 4-item Barthel index was determined by the physiotherapist on day 1, 5, 16 and 40. Three physiotherapists participated in the study. These were blinded towards the index procedure and applied the same physiotherapeutic regimen to all patienets in the study. Mobilization started on the day after the procedure under full weight bearing of the operated limb.

On day 1, 5, 16 and 40 after the procedure, the hemoglobin and the VAS were determined. Complications that necessitated surgical procedures or had the capability to prolong the hospital stay were noted. Statistical analysis, including study size determination based on the main outcome parameter (4-item Barthel index), was performed by a medical statistician. Non-parametric test methods including the Mann-Witney-U Test and the Friedman-Test were used. The level of significance was set as p < 0.05. For post-hoc power analysis the program G-Power, ver. 3.1.2, University Kiel, Germany) was used.

## Results

Evaluation of the Barthel index showed no significant differences between the study arms before the injury and at day one following surgery. On the remaining study time points there was a detectable significant difference indicating a higher score for the minimal invasive DAA group (Table 3). The VAS as a measurement of experienced pain did not show any significant differences between the study arms until day 16, from where on the DAA patients had a lower VAS score (for details see Table 3), indicating less pain as compared to group 2. There were no detectable differences in the requirements of packed red cells between the study arms over the study period.

**Table 3:** Results I

**Four-item Barthel**	**Pre fracture**	**Day 1**	**Day 5**	**Day 16**	**Day 40**
**Median**					
DAA	42.5 (5–50)	0 (0–20)	20 (0–50)	25 (5–50)	42.5 (5–50)
	StdDev.13.9	StdDev.5.8	StdDev.13.6	StdDev.13.1	StdDev.14.6
Watson-Jones	40 (25–50)	0 (0–15)	10 (0–5)	20 (0–45)	30 (5–45)
	StdDev.7.4	StdDev.5.4	StdDev.10.2	StdDev.13.9	StdDev.11.9
P-Value	p = 0.55	p = 0.47	**p = 0.009**	**p = 0.05**	**p = 0.013**
					**(1-β = 0.96)**
**Pain (VAS)**					
Median					
DAA	6 (0–8)	4 (1–8)	2 (0–5)	1 (0–5)	0 (0–1)
	StdDev.1.9	StdDev.1.96	StdDev.1.4	StdDev.1.33	StdDev.0.31
Watson-Jones	5.5 (0–9)	5 (2–8)	4 (0–5)	2 (0–4)	1 (0–2)
	StdDev.2.0	StdDev.1.66	StdDev.1.6	StdDev.1.53	StdDev.0.82
P-Value	p = 0.37	p = 0.88	p = 0.14	**p = 0.035**	**p = 0.0004**
**Packed red cells**	Min.	Mean	Max.	StdDev.	P-Value
**(Units)**					
DAA	0	1.1	4	1.4	
Watson-Jones	0	1.7	12	3.5	p = 0.44

Results of Barthel index, VAS pain scoring and packed red cell transfusion (during the study period).

Although 9.2 minutes longer in the DAA group, the theatre time (skin to skin time) was not significantly different in the two groups. The incision length was significantly shorter in the DAA group. In both groups the postoperative haemoglobin level was significantly lower than the preoperative level (p = 0.006), the postoperative haemoglobin levels did not differ significantly between the study arms (for details see Table 4).

**Table 4:** Results II

**Skin to skin time in min.**	**Mean**	**Min/Max**	**Std. Dev.**	**P-value**
DAA	**73.6 min.**	48/90	14.4	
WJA	**64.8 min.**	40/94	17.1	n.s.
**Incision length in cm**				
DAA	**8.0 cm**	6.5/13.5	1.5	
Watson-Jones	**12.4 cm**	8.0/15.5	1.5	0.0003
**Hämoblobin postop. g/l**				
DAA	**110.5**	76/138	16.3	
WJA	**105.0**	65/129.0	15.0	n.s.

Intra- and direct postoperative data. n.s. = not significant.

The radiographic evaluation showed a difference in the femoral offset of mean −3.2 mm for the DAA (SD −2.4) compared to 2.4 mm (SD 1.8) for the WJA. The difference was not statistically significant (p = 0.19, Mann–Whitney-U). Also no significant difference was found for the leg lenght with a mean difference of +3.8 mm (SD 2.5) for the DAA and + 3.1 (SD 1.7) for the WJA (p = 0.23, Mann–Whitney-U Test). A normal prosthesis alignment was determined for 77% after DAA and 71.5% after WJA.

### Complications during 40 day study period

There were no femoral shaft fissures or fractures in either group; also there were no detectable neurological lesions. Apart from the two excluded patients with dysplastic hips requiring a total hip arthroplasty, there were no dislocations in both groups. It was not necessary to convert from the minimal invasive to a conventional approach.

One patient in the DAA and two patients of the WJA group developed a deep vein thrombosis, no pulmonary embolism occurred during the study period. One patient in the WJA group developed an infected haematoma (ß-haemolysing streptococci) that resolved after surgical revision with implant in situ. One patient in the WJA group required twelve units of packed red cells in the 6 postoperative days, no surgical cause for this was found. One patient of the DAA group developed a wound edge necrosis at the proximal wound pole that resolved under conservative measures. There was no statistical significant difference regarding the complication rates.

The mortality was zero in the 40 day study period in both groups.

## Discussion

The mobilization process after bipolar hemi-arthroplasty can be improved, if a minimal invasive direct anterior approach is used as compared to a conventional approach.

There exist several studies comparing elective minimal invasive (MIS) hip arthroplasty with conventional approaches. The results of these studies regarding the clinical outcome, operating time and blood loss is often unequivocal. The outcome measurement systems used in these studies (e.g. Harris hip score [41]) were all developed for a much younger, mentally fit and active patient population. Also these scoring systems were mostly constructed for a long term monitoring of the results of hip surgery.

In a recent systemic literature review, 14 commonly used outcome scales were found to be used for patients with proximal femoral fractures [18]. None of these test methods were validated for use in this patient group. The author failed to identify a validated test method for this patient population. It remains unclear if the test methods that are used to detect the differences between minimal invasive and conventional hip arthroplasty (often HHS or WOMAC) might lack the discriminatory power in a short term study like ours; it is certain that these test methods are of limited use in a geriatric population. In a recent retrospective study, the period to successful mobilisation was measured in patients treated with a hemi-arthroplasty either with a conventional or a MIS approach [42]. Apart from the fact that this was a retrospective study, the time point of successful mobilization was defined as the date at which the patient was able to stand coordinated with both legs and under use of available walking aids lift the non-operated leg from the floor. This item appears methodically difficult, especially in a retrospective study.

Although not validated for patients with a fractured proximal femur, the Barthel index has been used in trials before [21-26].

For this study we decided to use an abbreviated version of the Barthel index. This method that has been validated in the past for geriatric neurological rehabilitation patients [27]. We decided to use such an abbreviated test method with a focus on lower extremity function measured by activities of daily ling (ADL) in a geriatric population to increase the discriminatory power. This method is certainly not hip specific and we did not have the possibility to validate it previous to our trial.

Our results show an equal result at day 1 after surgery with the lowest possible 4-item Barthel index of median 0. This is not surprising as these frail patients often only get mobilized briefly on that day with the help of two physiotherapists. On day 5 and the following measurements, the DAA group showed superior results regarding the mobilization process. In the only published randomized trial comparing minimally invasive versus conventional hemi-arthroplasty for femoral neck fractures, full weight bearing was achieved faster in the minimal invasive group [43]. Unfortunately this study neither specified the implants used nor how many were implanted cemented or uncemented.

Another randomized trial compared the mobility 48 hours after surgery and found no difference for the activities transfer from supine to sit, transfer from sitting to standing, mobilizing, ascending and descending stairs and weight-bearing for patients with elective THA [44]. A recent trial comparing minimal invasive and conventional approaches for THA distinguished between anterolateral and posterolateral approaches [45]. In this study, the superior results of the minimal invasive group regarding the HHS at 6 weeks were mainly found in the posterolateral access group. Other randomized trials failed to detect a significant difference in clinical outcome [46,47]. Two meta-analyses failed to detect a significant difference between minimal invasive and conventional elective THA regarding the HHS [16,48].

Operative time was about nine minutes longer for the minimal invasive group in our study, the difference between groups was not significant. Similar results have been reported before [43], although possibly the operating time is shorter in minimal invasive procedures using a posterior approach [16,48]. Unsurprisingly, the skin incision length in our study was shorter for the minimal invasive DAA group. We cannot fully exclude a bias as the length was only measured once.

A number of studies have measured intraoperative or postoperative blood loss [43,46,47,49,50], the results are mixed. Even three meta-analyses came to divergent findings on this topic, showing either a highly significant advantage for a minimal invasive procedure [15,16] or no significant difference [48]. As the intraoperative blood loss is very difficult to measure exactly, we decided to determine the postoperative haemoglobin level, which showed no difference between the study arms. Also the measurement of administered packed red cell units was equivalent between the groups.

We found no difference in the direct postoperative pain using the VAS. Interestingly there was a detectable difference with less pain in the DAA group from day 5 onwards, which was still measurable at the end of the study on day 40. As the difference was small, it remains unclear if it is clinically relevant. A better pain control in a minimal invasive group also has been found in elective THA [17].

The radiological analysis revealed no statistically significant differences between the two groups. Nevertheless no direct conclusions should be drawn from this fact for two reasons: First the study size was determined for the comparison of the 4-item Barthel index and it is possible that this prevented a difference from getting evident. Secondly the radiographic evaluation of plain radiographs for the measurement of hip arthroplasty is not very accurate [51,52]. As this is mainly caused by systematic errors, both groups should be influenced about equally.

According to the German “BQS national quality report in orthopaedics and traumatology”, a 30 day mortality rate of 5.9% has been reported in 45,051 patients treated with an endoprosthetic device for a fractured neck of femur in 2007 [53]. In our study with no mortality, frail and bedridden patients were excluded as the primary outcome item measured mobility. In a study with a similar patient collective, two of 69 patients died (2.8%) due to pulmonary embolism [54]. Regarding intra- and postoperative complications we found no evidence that a minimal invasive approach results in a higher complication rate. All surgeons involved in the treatment in our study were on a senior level with a long experience in hip fracture treatment; also they underwent cadaveric training by the inventors of this method previous to the trial. The results regarding intra- and postoperative complications might therefore not be directly applicable to various hospital settings. Complication rates of this approach have only been described for elective hip surgery [9-11], it is unlikely that these are lower in patients with a fractured neck of femur.

The strength of the present study is that it is a prospectively randomized trial with a single type of implant and a homogenous surgical method. Apart from the type of approach, the treatment scheme was identical and the population of the groups was comparable.

The present study is not able to address the question if an improved mobilisation results in fewer complications or a lower mortality, this is certainly a weakness. Much larger groups and a longer follow up period would be required to answer for this. There was no external monitoring of this study; as a result we only provided level 2 evidence with this trial.

## Conclusion

Our findings suggest that the mobilisation process in the first 40 days is favourable if a minimal invasive approach is used.

## Competing interests

No competing interests to declare, this research was funded by the University Lübeck.

## Authors’ contributions

FR, MW and AP developed the study idea, FR and AU finalized the study protocol and performed the radiological evaluation, SJ performed the clinical follow up studies, APS participated in its design and coordination and helped to draft the manuscript. All authors read and approved the final manuscript.

## Pre-publication history

The pre-publication history for this paper can be accessed here:

http://www.biomedcentral.com/1471-2474/13/141/prepub
